# A New Method for Constructing Atmospheric Plates

**Published:** 1868-08

**Authors:** N. T. Folsom

**Affiliations:** Boston.


### ARTICLE III.

### *A New Method for Constructing Atmospheric Plates.*

By N. T. Folsom, Boston.

I have a method of constructing the atmospheric plates of artificial teeth so that they will not move from their places in the mouth while eating or speaking, neither will food get under them, however difficult the mouth. I will describe it: First take an impression in wax and trim off the surplus, then press out the wax that comes against the labial surface of the dental ridge, to give room for the plaster, after which cool the wax. I now have a cup suited to the case, with which I take the impression in plaster, mixed with a solution of sulphate of potassa (Dr. Chamberlin's rule is ½ oz. to 1 qt. water,) varnish with ethereal varnish, mix the plaster for making the model the same as for the impression (with the solution of potassa,) and then dip the impression in water and immediately pour the plaster. The next step is to examine the mouth to ascertain where the edge of the plate will extend ; then mark that line on the model. Examine the mouth again to ascertain the yielding nature of the parts of this same line, note the hard and soft places, and then with a suitable instrument cut a grove in the plaster model along the entire line of the edge of the plate, one-twentieth of an inch wide, and varying in depth from one-sixteenth to one-sixtieth of an inch. As a general rule I commence cutting the groove in the rear of the tuberosity on the right side of the mouth. At this point I cut it deep, then shallow until I reach the soft part at the side of the back of the mouth, which I cut deeper than at any other point, then shallow again till I reach the corresponding point on the opposite side.

From the rear of the tuberosities to the canine teeth, I cut it comparatively shallow. I endeavor to cut a well defined groove around in front from canine to canlne, quite deep. A plate made on this cast if not injured in vulcanizing or otherwise, will fit not only the mouth, so that atmospheric pressure is obtained, but has a packing ridge encircling it which prevents ingress of air under it, and thereby secures the pressure on the outside. I make the ridge as high all around the plate as is possible taking care that it does not pinch the flesh on the bone; as it is not necessary to have it hurt at all to secure the plate firmly.

In finishing the plate I do not reduce the ridge in width or height, or break its continuity. When ready to put in, I let the patient carry it to its place and after wearing it ten or fifteen minutes, if the ridge is hurting in any place, I reduce it at the point indicated, by scraping it a little until the plate sets easily. There are points along the edge of the plate where the under surface would need reducing, if no ridge was present; I reduce it and the ridge correspondingly. If after the plate has been worn a day or two, some points are bearing too hard, I reduce the ridge at those points. I had mnch rather have a patient come in and say that the plate is firmly fixed in the mouth, but is hurting at some point, than to have them say, they can do nothing with them, because in the first case I can correct it, but in the last I have trouble. The reason why a plate made in this way is so firmly secured, is well set forth by Dr. Davis of New Bedford, when he says there is a movement of the plate attending mastication, and the least lifting admits the air and down comes the plate, whereas the packing ridge allows of this movement without admitting the air.

